# One-year survival rate and healthcare costs after cardiac arrest in Taiwan, 2006–2012

**DOI:** 10.1371/journal.pone.0196687

**Published:** 2018-05-01

**Authors:** Yi-Ming Weng, Chip-Jin Ng, Chen-June Seak, Cheng-Yu Chien, Kuan-Fu Chen, Jr-Rung Lin, Chee-Jen Chang

**Affiliations:** 1 Department of Emergency Medicine, Prehospital Care Division, Tao-Yuan General Hospital, Tao-Yuan, Taiwan; 2 Department of Emergency Medicine, Chang Gung Memorial Hospital, and Chang Gung University College of Medicine, Linkou, Taiwan; 3 Faculty of Medicine, National Yang-Ming University School of Medicine, Taipei, Taiwan; 4 Department of Emergency Medicine, Ton-Yen General Hospital, Zhubei City, Hsinchu county, Taiwan; 5 Department of Emergency Medicine, Chang Gung Memorial Hospital, Keelung, Taiwan; 6 Community Medicine Research Center, Chang Gung Memorial Hospital, Keelung, Taiwan; 7 Clinical Informatics and Medical Statistics Research Center and Graduate Institute of Clinical Medicine, Chang Gung University, Tao-Yuan, Taiwan; 8 Department of Anesthesiology, Chang Gung Memorial Hospital, Tao-Yuan, Taiwan; 9 College of Medicine, Chang Gung University, Tao-Yuan, Taiwan; 10 Research Services Center for Health Information, Chang Gung University, Tao-Yuan, Taiwan; 11 Department of Cardiovascular Medicine, Chang Gung Memorial Hospital, Tao-Yuan, Taiwan; University Medical Center Goettingen, GERMANY

## Abstract

**Objectives:**

The annual increase in costs and the quality of life of survivors of cardiac arrest are major concerns. This study used National Health Insurance Research Database (NHIRD) of Taiwan to evaluate the 1-year survival rate and the annual healthcare costs of survivors after cardiac arrest.

**Methods:**

This retrospective, fixed-cohort study conducted from 2006 to 2012, involved 2 million individuals randomly selected from the NHIRD of Taiwan. Adult patients at least 18 years old who were diagnosed with cardiac arrest were enrolled. Survival was followed up for 1 year.

**Results:**

In total, 2,256 patients were enrolled. The survivor cohort accounted for 4% (89/2256) of the study population. There were no significant differences in the demographic characteristics of the survival and non-survival cohorts, with the exceptions of gender (male: survival vs. non-survival, 50.6% vs. 64.5%, p = 0.007), diabetes mellitus (49.4% vs. 35.8%, p = 0.009), and acute coronary syndrome (44.9% vs. 31.9%, p = 0.010). Only 38 (1.7%) patients survived for > 1 year. The mean re-admission to hospital during the 1-year follow up was 73.5 (SD: 110.2) days. The mean healthcare cost during the 1-year follow up was $12,953. Factors associated with total healthcare costs during the 1-year follow up were as follows: city or county of residence, being widowed, and Chronic Obstructive Pulmonary Disease (city or county of residence, β: -23,604, p < 0.001; being widowed, β: 25,588, p = 0.049; COPD, β: 14,438, p = 0.024).

**Conclusions:**

There was a great burden of the annual healthcare costs of survivors of cardiac arrest. Socioeconomic status and comorbidity were major confounders of costs. The outcome measures of cardiac arrest should extend beyond the death, and encompass destitution. These findings add to our knowledge of the health economics and indicate future research about healthcare of cardiac arrest survivors.

## Introduction

Although the survival rate of cardiac arrest patients varies geographically, there is a trend towards improvement following investment in pre-hospital and hospital settings [1–4]. Studies had addressed the issue of unlimited resuscitation efforts without evaluation of the appropriateness of diverting medical resources, which results to financial pressure as a burden on healthcare system [5,6]. It has been proposed that resources be shifted away from the provision of futile medical procedures. While advanced cardiac life support is considerable cost [5], the annual increase in costs and the quality of life of survivors of cardiac arrest are major concerns [7,8]. Instead of assessing costs until hospital discharge after cardiac arrest, the annual healthcare costs of survivors should be considered under the assumption that survivors enjoy a good quality of life [9]. Although there were studies reporting the healthcare costs of cardiac arrest survival using different databases[7,8,10,11], limited data were available in such a universal health care coverage healthcare system.

This study used National Health Insurance Research Database (NHIRD) of Taiwan to evaluate the 1-year survival rate and the annual healthcare costs of survivors after cardiac arrest as well as to identify the associated factors.

## Materials and methods

### Study design

This retrospective, fixed-cohort study conducted from 2006 to 2012, involved 2 million individuals randomly selected from the NHIRD of Taiwan. The NHIRD is representative of the entire population with insurance coverage (*i*.*e*., 99% of the 23 million people in Taiwan)[12]. The NHIRD is an electronic database developed by the NHI administration and the Ministry of Health and Welfare (MOHW), Taiwan, and has been described and validated [13–15]. There was no risk of loss of privacy. All the individual information data must be in independent work area and in the data received the patients' ID is masked by MOHW. The results with all analysis table or figure must be consistent with the research purpose and must be limited to statistics from more than three subjects. These results were reviewed and approved by MOHW. This study was approved by the Ethics Committee of Chang Gung Medical Foundation and the Ministry of Health and Welfare, Taiwan. The Ethics Committee of Chang Gung Medical Foundation and the MOHW, Taiwan waived the requirement for informed consent.

The study protocol is provided at http://dx.doi.org/10.17504/protocols.io.naddaa6.

### Study population

Patients who were diagnosed with cardiac arrest with an International Classification of Diseases, Ninth Revision (ICD-9) code of 427.5 combined with a medical procedure code of 47029C (cardiopulmonary resuscitation at ED) were enrolled. Patients younger than 18 years old and uncertain cases not admitted after the index event for whom only outpatient records were available were excluded.

### Study cohorts

The study population was divided into death in hospital and survival to discharge cohorts. The cohort with death in hospital comprised patients without medical records in the NHIRD after the day of the ED visit or who were discharged from inpatient care. The retrieved dates of death were verified using the death register. The cohort with survival to discharge comprised patients not included in the cohort with death in hospital and those with medical records for 1 year of follow-up available in the NHIRD. The cohort with survival to discharge was followed up for 1 year, and reported as the time elapsed from the day of ED visit until the date of death according to the death register or survival status at 1 year after cardiac arrest.

### Main outcome measurements

The main outcome measurements were the total healthcare costs (in US dollars [$]) of survivors during 1 year of follow up, including outpatient, inpatient, and ED costs. The average exchange rate during the study period was 31.5 Taiwan New Dollars per $1. Factors associated with healthcare costs were identified. Additionally, the costs of resuscitation in the ED and further inpatient care were evaluated.

### Covariate assessment

The following socioeconomic variables were analyzed: area of residence (municipalities directly under the central government vs. cities or counties), marital status (single, married, divorced, widowed), and education level (junior high school or higher vs. others). In terms of comorbidities, the following conditions included in the Charlson Comorbidity index were analyzed [16]: diabetes mellitus (ICD-9 code: 250), hypertension (401–405), acute coronary syndrome (410–414), heart failure (428), cerebrovascular accident (433–437), chronic obstructive pulmonary disease (COPD) (490–496, 500–508), liver cirrhosis (571.2, 571.5), renal failure (585, 586, 588, 58001–58030), and malignant neoplasms and malignant lymphatic/hematopoietic neoplasms (140–199, 200–208). Procedures performed during resuscitation in the ED—such as cardioversion (47028C), percutaneous coronary intervention (PCI; 36.0–36.03, 36.05–36.09), intra-aortic balloon pumping (IABP; 97.44), pacemaker implant (37.8), coronary artery bypass graft (CABG; 36.1–36.99), and blood transfusion (94001C,94002C,94013C,94015C,94003C)—were analyzed. The type of hospital (medical center vs. regional hospital) was included in the analysis.

### Statistical analysis

The demographic characteristics of the survival and non-survival cohorts were compared, and the costs of ED visits and any inpatient care were calculated. A survival curve of the study cohort during the 1-year follow-up period was generated using the Kaplan–Meier method. Linear regression was conducted to identify the associations of various factors with 1-year healthcare costs after adjusting for age and sex.

The data were analyzed using SAS 9.4 (SAS Institute Inc., Cary, NC, USA). Categorical variables are presented as count and percentages and were compared using the chi-squared test or Fisher’s exact test. Continuous variables are presented as means and standard deviations (SDs) and were compared using Student’s *t*-test. A *p-*value < 0.05 was considered to indicate significance.

## Results

### Demographic characteristics

**Fig 1:**
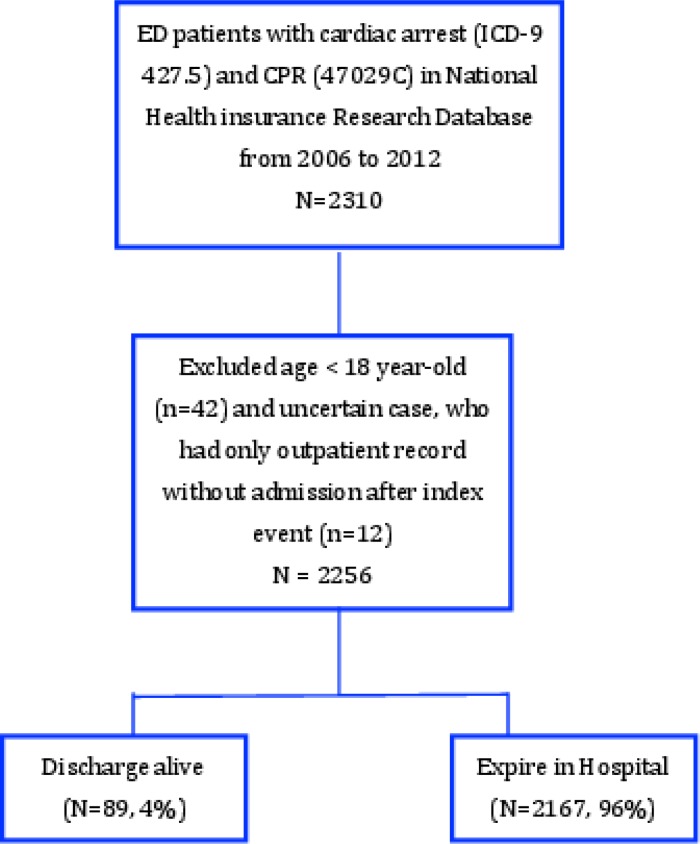
Patients enrolled.

In total, 2,256 patients with ICD-9 code 427.5 and procedure code 47029C in the ED were enrolled after excluding 42 patients younger than 18 years old and 12 uncertain cases for whom only outpatient records were available (Fig 1). The survivor cohort accounted for 4% (89/2256) of the study population. Among the non-survivor cohort, 2,167 (96%) patients were pronounced dead in the ED.

Table 1 shows the demographic characteristics of the study population. Males predominated (64%), and the mean age was 67.7 years. Of the participants, 20.7% lived in municipalities directly under the central government; 37.9% had a junior high school or higher educational level, and 63.5%, 12.2%, 7.2%, and 16.9% were married, single, divorced, and widowed, respectively. Hypertension was the most common comorbidity (62.8%), followed by diabetes mellitus (36.3%) and acute coronary syndrome (32.4%). There were no significant differences in the demographic characteristics of the death in hospital and survival to discharge cohorts, with the exceptions of gender (male: survival to discharge vs. death in hospital, 50.6% vs. 64.5%, p = 0.007), diabetes mellitus (49.4% vs. 35.8%, p = 0.009), and acute coronary syndrome (44.9% vs. 31.9%, p = 0.010). Less than 10% of cardiac arrest patients were resuscitated at ED of a medical center, and 16.6% underwent cardioversion in the ED. Patients in the cohort with survival to discharge underwent a greater number of procedures in the ED than did those in the cohort with death in hospital.

**Table 1:** Demographic characteristics of study cohorts.

	ALL	Death in Hospital	Survival to Discharge	p-value
	(N = 2256)	(N = 2167, 96%)	(N = 89, 4%)	
Age in years, mean (SD)	67.7(17.9)	67.7(18.0)	68.2(15.9)	0.763
Male, N (%)	1443(64.0)	1398(64.5)	45(50.6)	0.007
Place of residence, N (%)				
Municipalities	467(20.7)	447(20.6)	20(22.5)	0.674
City or county	1789(79.3)	1720(79.4)	69(77.5)	
Marriage, N (%)*(3missing)				
Single	276(12.2)	271(12.5)	5(5.6)	0.225
Married	1433(63.5)	1376(63.5)	57(64.0)	
Divorced	162(7.2)	154(7.1)	8(9.0)	
Widowhood	382(16.9)	364(16.8)	18(20.2)	
Education level, N (%)*(30missing)				
Junior high school graduated and/or higher education	854(37.9)	822(37.9)	32(36.0)	0.757
Comorbidities, N (%)				
Diabetes mellitus	820(36.3)	776(35.8)	44(49.4)	0.009
Hypertension	1417(62.8)	1354(62.5)	63(70.8)	0.112
ACS	731(32.4)	691(31.9)	40(44.9)	0.010
Heart failure	509(22.6)	485(22.4)	24(27.0)	0.311
CVA	588(26.1)	562(25.9)	26(29.2)	0.490
COPD	865(38.3)	834(38.5)	31(34.8)	0.487
Renal failure	337(14.9)	317(14.6)	20(22.5)	0.042
Malignancy	344(15.2)	334(15.4)	10(11.2)	0.283
Medical center as receiving hospital, N (%)	207(9.2)	197(9.1)	10(11.2)	0.492
Procedure at ED				
Cardioversion, N (%)	383(17.0)	360(16.6)	23(25.8)	0.023
Percutaneous coronary intervention (PCI)	213(9.4)	189(8.7)	24(27.0)	< .0001
Intra-aortic balloon pumping (IABP)	28(1.2)	21(1.0)	7(7.9)	< .0001
Pacemaker implant	54(2.4)	46(2.1)	8(9.0)	< .0001
Open heart surgery**	42(1.9)	37(1.7)	5(5.6)	0.022
Blood transfusion	963(42.7)	905(41.8)	58(65.2)	< .0001

Abbreviations: SD, Standard deviation; ACS, Acute coronary syndrome; CVA, cardiovascular accident; COPD, chronic obstructive pulmonary disease; ED, emergency department; OP, operation

**Open heart surgery including coronary artery bypass graft, aorta surgery, and other open heart surgery

### Survival duration and total healthcare costs

**Fig 2:**
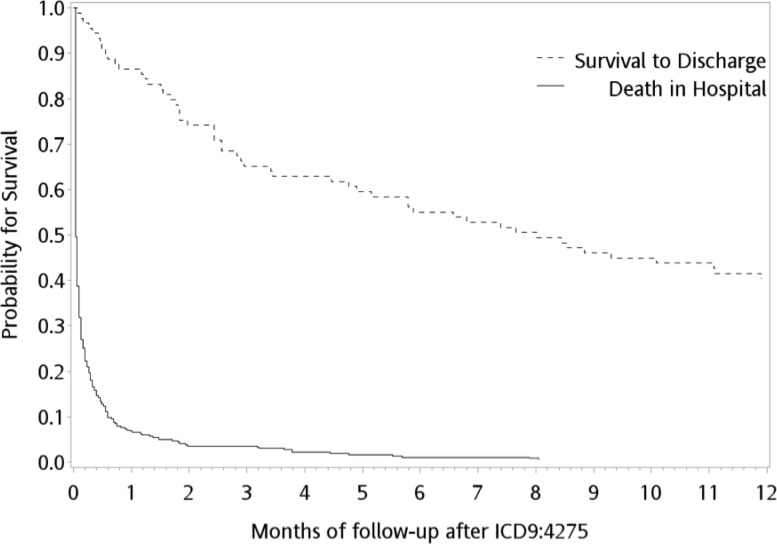
The survival curve demonstrated probability of survival to discharge over months between two cohorts.

The survival curve is shown in Fig 2. The mean survival duration during the 1-year follow up was 10 ± 53 days overall and 217 ± 146 days in group for survival to discharge. Only 38 (1.7%) patients survived for > 1 year. In group for survival to discharge, the mean number of outpatient visits, ED visits, and admissions during the 1-year follow up were 14, 1, and 1, respectively. The mean hospital stay duration after index event and re-admission to hospital during the 1-year follow up was 30 (SD: 22) and 73.5 (110.2) respectively.

Table 2 shows the healthcare costs of the study cohorts. The mean costs of medical care for cardiac arrest until discharge in the death in hospital and survival to discharge cohorts were $522 and $18,859, respectively. The mean healthcare cost during the 1-year follow up was $12,953, and the costs for inpatient care accounted for the majority of this amount. Factors associated with the total healthcare costs of the cohort with survival to discharge are shown in Table 3. A multivariate analysis of factors associated with total healthcare costs during the 1-year follow up after adjusting for age and gender were as follows: city or county of residence, being widowed, and COPD (city or county of residence, β: -23,604, p < 0.001; being widowed, β: 25,588, p = 0.049; COPD, β: 14,438, p = 0.024).

**Table 2:** The health care costs of study cohorts.

	All	Death in Hospital	Survival to Discharge
	(N = 2256)	(N = 2167, 96%)	(N = 89, 4%)
**Health care costs till discharge (USD)**			
Mean (SD)	1,247 (8,459)	522 (1,447)	18,895 (38,131)
**Total health care costs of survivors during one year follow-up (USD)**			
Mean (SD)			12,953 (27,726)
Out-patient department			1,551 (3,728)
Emergency department			204 (431)
In-patient department			11,589 (28,011)

Abbreviation: SD, Standard deviation

**Table 3:** Factors associated with healthcare costs of survivors during follow up.

	Sex-age adjusted model (USD)						
	β	95%CI					p
**Place of residence (vs Municipalities)**	-23,604	(	-36,664	to	-10,543	)	<0.001
**Marriage (vs divorced)** City or County							
Single	3,420	(	-29,284	to	36,124	)	0.838
Married	13,234	(	-8,076	to	34,545	)	0.224
Widowhood	25,588	(	141	to	51,036	)	0.049
**Education level (vs others)**							
Junior high school graduated and/or higher education	3,283	(	-10,248	to	16,814	)	0.634
**Comorbidities, N(%)**							
Diabetes mellitus	-11,343	(	-22,864	to	177	)	0.054
Hypertension	6,671	(	-6,502	to	19,844	)	0.321
ACS	-3,779	(	-15,719	to	8,161	)	0.535
Heart failure	8,491	(	-4,823	to	21,805	)	0.211
CVA	-4,989	(	-18,140	to	8,162	)	0.457
COPD	14,438	(	1,944	to	26,933	)	0.024
Renal failure	-2,746	(	-20,836	to	15,344	)	0.766
Malignancy	-531	(	-14,652	to	13,589	)	0.941
**Procedure at ED**							
Cardioversion, N (%)	-236	(	-13,473	to	13,002	)	0.972
Percutaneous coronary intervention (PCI)	-6,181	(	-19,292	to	6,931	)	0.356
Intra-aortic balloon pumping (IABP)	-4,794	(	-26,489	to	16,901	)	0.665
Pacemaker implant	-7,953	(	-28,085	to	12,179	)	0.439
Open heart surgery**	9,155	(	-15,799	to	34,109	)	0.472
Blood transfusion	3,761	(	-8,394	to	15,916	)	0.544
**Status at ED**							
Healthcare costs till discharge	0.0126	(	-0.0007	to	0.0259	)	0.063
Length of stay till discharge	238	(	-45	to	520	)	0.099

Abbreviations: CI, confidence interval; ACS, Acute coronary syndrome; CVA

cardiovascular accident; COPD, chronic obstructive pulmonary disease; ED, emergency department

**Open heart surgery including coronary artery bypass graft, aorta surgery, and other open heart surgery

## Discussion

We evaluated the 1-year survival rate and related healthcare costs after cardiac arrest in a population randomly sampled from the NHIRD, Taiwan. The rates of survival to discharge and after 1 year were 4% and 1.7%, respectively. In the Pan Asia Outcome Study, the rates of overall survival to hospital discharge were 0.5–8.5% [17]. In the Japan national administrative claims data from 2008 to 2009, the rate of survival to discharge of out-of-hospital cardiac arrest was 7.6% [10], and that in Jerusalem from 2005 to 2010 was 6.1% [11]. Our results showed that the mean cost per hospital discharge after cardiac arrest was less than $32,000, whereas it was $8,000 to $50,000 in previous studies performed in diverse locations [8,10,11,18]. The discrepancies might due to differences in healthcare payments or performance of different medical procedures during the post-resuscitation care provided by different systems. In a retrospective survey in a Dutch university hospital, Walchelder *et al*. reported that PCI and therapeutic hypothermia contributed to long-term function [19]. In our study, 27% and 8.7% of death in hospital and survival to discharge cohorts, respectively, underwent PCI, and the mean healthcare cost during the 1-year follow-up period was ~ $13,000. Graf *et al*. reported a total healthcare cost of $10,107 per life-year gained, which was ~ 78% of that in this study [8]. According to our results, during the 1-year follow up, survivors repeatedly visited the hospital, and their stays tended to be prolonged. Inpatient healthcare accounted for the majority of the healthcare costs; the average length of re-admission to hospital stay was 73.5 days during the first year of life. Therefore, the survival rate in study area was low, and healthcare costs were high during the 1-year follow-up period.

Survival with a cerebral performance category of 1 or 2 is an important outcome in cardiac arrest patients [20]. Indeed, it is reasonable to question the quality of life and the ability to perform the activities of daily living of individuals who have undergone long hospital stays during their first year of survival. Implementation of Chain of Survival effectively improved outcomes of ventricular fibrillation arrests[21]. The reallocation of resources to facilitate the links in Chain of Survival yield better survival rate and improve functioning and quality of life. Cardiac arrest survival and medical resource usage are not only outcomes but also may guide planning with regard to healthcare resource allocation and policy.

Several factors were associated with the healthcare costs of survivors after adjustment for age and sex; these were place of residence, being widowed, and COPD. Comorbidities are predictive of early mortality and re-admission after discharge. The LACE comorbidity index includes length of stay, acuity of admission, comorbidity (Charlson score), and ED use in the previous 6 months [22]. Although previous studies did not focus on cardiac arrest survivors after discharge [23,24], cardiac arrest patients showed a high acuity of admission and ED use after index event. Use of a multi-comorbidity model would enable a more detailed analysis of disease burden. COPD was the only comorbidity associated with the healthcare costs of cardiac arrest survivors in this study.

Socioeconomic status and household income are associated with healthcare costs. Previous studies in Taiwan revealed an unequal geographical distribution of medical care resources and healthcare coverage. Bikdeli *et al*. reported that the neighborhood level predicts re-admission of heart failure patients [25]. One study reported that the outcomes of patients with large B-cell lymphoma differed between urban and rural areas, and another found that treatment and outcomes disparities in lung cancer [26,27]. In general, persons in rural areas experience more difficulty obtaining medical treatment with minimal delay of emergency medical service. However, the survival rate showed no difference between city/county and municipalities in this study. In contrast, healthcare costs of cardiac arrest survivors were significant lower for residents of city/county than municipalities. Instead of distribution of medical resource and payments, different goal and mode of care for cardiac arrest survivors across the metropolis and city might contribute to the study results.

This study identified that marital state, as being widowhood, was associated with healthcare costs of cardiac arrest survivors. Widowhood is considered to reduce access to healthcare. Rolden *et al*. discovered that death of a spouse is associated with an increase in healthcare costs in the Netherlands by analyzing matched regional healthcare insurance and marital status data [28]. Moreover, loss of informal care was associated with increased long-term care expenses [29,30]. In addition to healthcare, this group requires integrated social care. This study indicates the associations of healthcare costs with place of residence and widowhood among cardiac arrest survivors in a universal coverage healthcare system. Policy maker should look into the difference of the healthcare costs of cardiac arrest survivors with universal coverage.

### Limitations

There are several limitations to the present study. First, several confounders were not evaluated, including the costs of emergency medical service system activation, home care, and long-term care, as well as out-of-pocket payments. Coding errors and omission of healthcare costs may have existed, and these would lead to underestimation of healthcare costs. However, we believe that the majority of costs of medical treatment were evaluated in this study. Furthermore, the cause of death (*e*.*g*., trauma, cardiac origin, drowning, *etc*.) was not determined.

Second, selection bias is possible due to the limited sample size and short study period. This study involved 2 million individuals randomly sampled from the NHIRD, which has been described and has been validated as representative of the population of Taiwan. We believe that the data is representative and the selection bias could be omitted.

Third, the study setting and population may limit the applicability of the findings.

The study population included both in-hospital cardiac arrest and out-of-hospital cardiac arrest patients, who were resuscitated at the ED. The origin of patients was not determined. Furthermore, the termination of resuscitation guide was insufficient in Taiwan, except for patients who had signed do-not-resuscitate forms or who presented with signs of death, such as decapitation, incineration, decomposition, or rigor mortis. All other patients underwent resuscitation and were transported to the ED. Pre-hospital pronouncement of death was less costly [31]. On the other hand, the different mode of healthcare insurance at different region also limits the generalizability of the findings.

## Conclusion

There was great burden of the annual healthcare costs of survivors of cardiac arrest. Socioeconomic status and comorbidity were major confounders of costs. The outcome measures of cardiac arrest should extend beyond the death, and encompass destitution. These findings add to our knowledge of the health economics and indicate future research about healthcare of cardiac arrest survivors.
